# Mammary Epithelial Cell Lineage Analysis via the Lyon's Hypothesis

**DOI:** 10.23937/2469-570x/1410018

**Published:** 2016-01-03

**Authors:** Andrea L. George, Gilbert H. Smith

**Affiliations:** Mammary Stem Cell Biology Section, BRL, CCR, NCI, NIH, Bethesda, MD 20892, USA

**Keywords:** Lineage Tracing, Mammary, Lyon's Hypothesis, X chromosome

## Abstract

Implants of mammary glands from a single mammary fat pad in a H253 transgenic female mouse heterozygous for a lacZ-labeled X chromosome were analyzed at various time points following transplantation into the epithelium-cleared mammary fat pads of immune-compromised mice. The results show that the lacZ-marked X chromosome, demonstrated by nuclear-associated X-gal staining, was confined to a single epithelial clone that gave rise to the cap cells of all growing terminal end buds (TEB) in the expanding mammary outgrowths and also the basal cells of the elongated ducts. The nuclei of luminal cells in these ducts were uniformly negative for lacZ expression indicating that they were derived from cellular precursors that contained a silenced lac-Z marked X chromosome. This observation confirms the earlier work of Williams and Daniel, who concluded that cap cells were the precursors of the basal (myoepithelial cells) of the subtending mammary ducts.

## Introduction

Several years ago, we showed that fully differentiated mammary outgrowths at parturition could compromise the epithelial progeny from a single mammary cell and proved that these were clonal by retroviral insertion within their somatic DNAs [1]. Subsequently, we demonstrated that the clonal nature of such outgrowths could be maintained through five serial transplantation passages even though partial senescence was observed [2]. In the present work epithelial lineage marking is demonstrated in mammary transplant outgrowths from a single female mouse bearing a single X chromosome bearing a beta-galactosidase (lacZ) reporter gene located in the silenced portion of this chromosome. Because in female mammals, one of the two X chromosomes is randomly inactivated in tissues during embryogenesis [3], we are able to visualize the mosaicism of X-inactivation during the recapitulation of mammary ductal growth in an epithelium-divested mammary fat pad in vivo. By way of explanation, all portions of adult mouse mammary epithelium are capable of complete regeneration of a functional mammary epithelial gland upon transplantation into an epithelial-divested mammary fat pad. The resulting outgrowths follow the identical developmental stages that occur in the intact post-pubertal mammary gland. Therefore we sampled 4 outgrowths at 4 weeks post transplant into pre-pubertal female hosts to observe the early stages of mammary ductal growth (lots of TEBs) and at 10 weeks (completely filled with ducts and no TEBs) to evaluate where X chromosome inactivation had occurred and in what epithelial cell types. In the present case we confirm the earlier work by Williams and Daniel [4] that postulated that cap cells at the tip of growing mammary ducts in vivo give rise to both body cells in the terminal end buds and myoepithelial cells at the outer edge of the subtending ducts.

## Materials and Methods

### Mammary fat-pad clearing

Female Athymic NCR Nu/Nu mice were used for transplantation studies. The surgical procedures for clearing the mammary epithelium from the #4 inguinal fat pads of 3 week-old female mice and the method of implanting either tissue fragments or cell suspensions have been described in detail in earlier publications [5-10]. Generally, the surgical procedures required to remove the host epithelium from the fat pads were performed immediately prior to insertion of the transplant or inoculation of cultured cells.

### Mammary tissue transplantation

Random fragments (∼1.0 mm^3^) of mammary epithelium (n = 20) were taken from a single virgin female H253 mouse mammary gland from those that were generously gifted by Dr. Vicki Hammond (University of Melbourne) and used for transplantation studies. The fragments were implanted as described above and the implanted glands as well as host glands were taken at various times post-op.

### Preparation of Mammary Gland Whole Mounts and X-gal Staining

Briefly, the number #4 inguinal fat pads were excised from the transplant-bearing mice and spread onto glass slides. The glands were spread to expose as much area to the glass and to fatten the sample to improve viewing. The glands were then fixed in Carnoy's fixative (1:3:6 ratio of acetic acid, chloroform and ethanol) for 4 hours at room temperature. They were then stained with carmine alum, dehydrated through a series of alcohols, cleared in xylene, and sealed with Permount and a glass coverslip. Whole mounts of inguinal glands containing lacZ+ cells were fixed and stained as previously described [6,11]. Briefly, whole inguinal fat pads were mounted on glass slides and fixed in 4% paraformaldehyde for 2 hours, permeabilized in 0.02% NP-40, 0.01% sodium deoxycholate, and 0.002 M MgCl_2_ in phosphate-buffered saline (1× PBS) overnight at 4°C and processed for X-gal as previously described [12]. For X-gal controls intact host gland were treated identically. Stained glands were repeatedly rinsed in 1× PBS, then post fixed with Carnoy's fixative. Glands were then dehydrated in a graded series of alcohol and cleared in xylenes before analysis.

### Immunohistochemistry

For histological examination, X-gal + glands were embedded in parafin and cut in 5 μm sections and mounted on positively charged slides. Sections from samples were counter stained with nuclear fast red to identify lacZ+ structures. Sections were subsequently cleared in xylenes and rehydrated through ethanol gradients. Antigen retrieval was performed by heating slides in a boiling water bath for 20 min. in Tris-EDTA pH 9.0 (Dako). Using 3% hydrogen peroxide for 15 minutes at room temperature removed endogenous peroxidase activity. Slides were blocked with normal horse serum (Vector Laboratories, Burlingame, CA) for 1 hour at room temperature and then incubated overnight with primary antibodies to smooth muscle actin (Zymed) or progesterone receptor (Dako) at 4°C. Secondary antibody staining was performed using the R.T.U. Vectastain (goat anti-rabbit/mouse) kit (Vector Laboratories). Staining was visualized using the DAB peroxidase substrate kit (Vector Laboratories) per manufacturer's recommendations. Slides were counterstained with Mayer's hematoxylin (Sigma-Aldrich) and negative tissue controls were included in all immunohistochemical analyses.

## Results and Discussion

**Figure 1:**
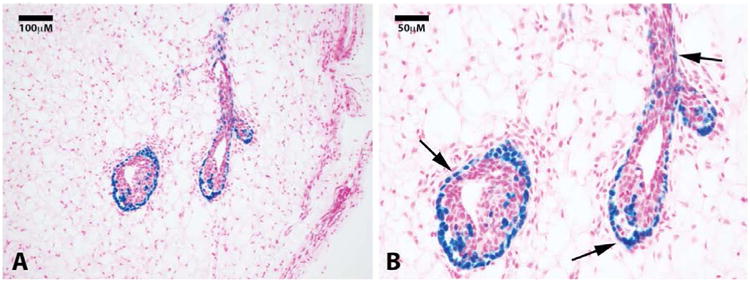
Clonogenic demonstration of labeled mammary cap cells and progeny. Photomicrographs of a nulliparous gland 4 weeks after H253 implantation show positive lacZ staining in the cap cells of terminal end buds at 20× (**A**) and 40× (**B**) magnification. Sections were counterstained with nuclear fast red. Black arrows point to positive cells in the basal epithelial layers.

**Figure 2:**
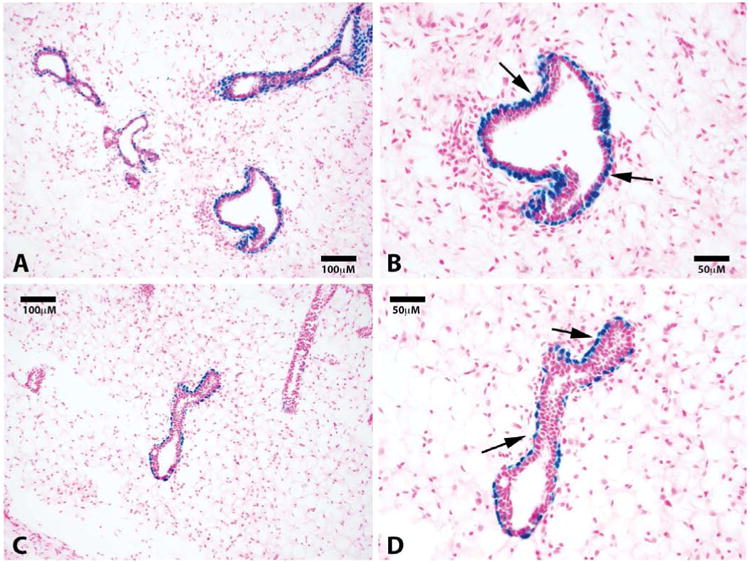
Luminal cells along subtending ducts are derived from a separate lacZ negative progenitor. Representative photomicrographs of mammary ducts at 20× (**A**) and 40× (**B**) show positive staining for lacZ cells in the myoepithelial cells along the basal surface. No luminal lacZ positive cells are found. Sections were counterstained with nuclear fast red. Black arrows point to lacZ positive basal cells.

**Figure 3:**
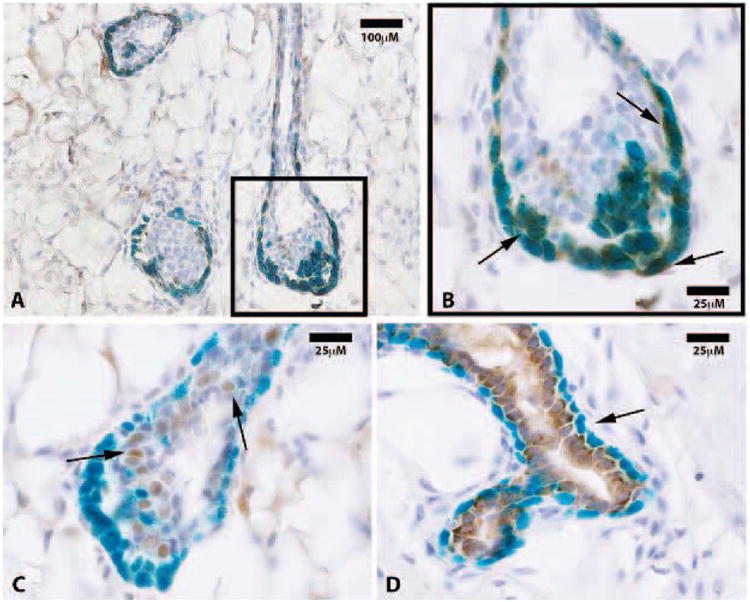
(**A**) LacZ positive progeny are positive for the myoepithelial marker smooth muscle actin (SMA) only. Representative immunohistochemistry section at 20× of mammary tissue stained for smooth muscle actin (SMA); (**B**) SMA staining co-localized with lacZ positive cap cells as well as those along the basal surface of subtending ducts (black arrows at 100×); (**C**) Representative immunohistochemistry section of mammary tissue stained for progesterone receptor (PR). PR positive cells were mostly negative for lacZ expression (100×, black arrows); (**D**) Photomicrograph of a mammary duct from a 10 week outgrowth at 100× shows cytokeratin 8 staining of lacZ-negative cells only. Black arrows point to lacZ positive, cytokeratin 8 negative myoepithelial cells.

Here, we take advantage of a transgenic model (H253) developed by Tam and Tan [13], where lacZ under the control of a ubiquitously active promoter (HMG CoA reductase) is inserted into the portion of the X chromosome that is inactivated during “lyonization” in random somatic cells during embryogenesis. We obtained mammary tissue from a heterozygous H253 female where only one of the two X chromosomes was marked by the transgene. We reasoned that individual fragments of this female's mammary gland would contain somatic cells in which one or the other X chromosome was silenced. Therefore random individual fragments from one of these mammary glands were transplanted into epithelium-divested mammary fat pads of immune-compromised female mice (Nu/Nu). From these implants, individual mammary outgrowth were expected and these would contain both cells in which the marked X chromosome was silenced and those in which the unmarked X was silenced; the latter would possess beta-galactosidase (X-gal+) positive nuclei. In 1 week (n = 4) and 4 week (n = 4) outgrowths we counted 19 TEB, all had associated cap cells which expressed lacZ. The body cells of the TEBs were negative for lacZ except for groups of contiguous lacZ-positive cells that were either responsible for the lacZ-positive cap cells or derived from them. None of the luminal cells associated with the subtending ducts were positive for lacZ including those found in completed outgrowths (shown at ten weeks, n = 8) Only foci of cells in the body of terminal end buds were positive for lacZ, as were the cap cells associated with these terminal buds and the basal myoepithelial cells lining the subtending duct (Figure1A and Figure 1B). This result confirms and extends the observations reported by Williams and Daniel [4], who concluded using different methods that the cap cells gave rise to the myoepithelial cells lining the subtending ducts. These authors also postulated that cap cells migrated into the body of the terminal end bud (TEB) where they potentially served as stem/progenitors of the luminal epithelium in the growing ducts. Our result suggests that the cap cells are produced by specific precursor cells in the body of the end buds and these do not play a role in the production of the luminal cell component of the ducts, which remain free of lacZ-positive nuclei during extension of the growing ducts at 4 weeks (Figure 2A and Figure 2B). Previous work from our laboratory [14] demonstrated the presence of duct-limited and lobulo-alveolar limited progenitors among nulliparous mammary epithelial cells using limited dilution transplantation studies. We confirmed this later [2] when we transplanted the clonal mammary outgrowths serially. These studies showed that ductal growth and lobulo-alveolar development decayed independently from one another in subsequently impregnated hosts. The present observation supports the presence of ductal-limited progenitors that give rise to the specialized cap cells, which are indispensable to branching ductal morphogenesis. These are lineage mapped by lacZ, which is the result of silencing of the unmarked X chromosome and reveals the clonal nature of the creation of the TEB-associated cap cells and the myoepithelial cells lining the basal portions of the extending mammary duct. Histological sections were also taken from 10 week outgrowths (N = 8) from fragments transplanted from the same mouse, no lacZ-positive luminal cells were found in any of these indicating that only the ductal progenitor was marked and only myoepithelial cells associated with the formed ducts or cells found in the body of the TEBs were lacZ-positive (Figure 2C and Figure 2D). To confirm that the lacZ-positive cells in the subtending ducts were differentiated myoepithelial cells, they were immuno-stained for smooth muscle actin (SMA), which is a lineage marker for mammary duct-associated myoepithelial cells (Figure 3A and Figure 3B). Staining demonstrated that indeed lacZ-positive cells expressed SMA while lacZ-negative luminal cells did not, instead expressing hormone receptor markers including progesterone receptor (Figure 3C). Further, staining of 10-week samples demonstrated that only lacZ-negative cells express the luminal marker cytokeratin 8 whereas lacZ-positive cells were cytokeratin 8 negative (Figure 3D).

Any portion of the mammary epithelium in the mouse, regardless of age or reproductive history, is capable upon transplant, to recapitulate an entire functional mammary gland in the epithelium-cleared mammary fat pad [15]. The results of our study indicates that a single clone (marked by an X chromosome carrying a lacZ gene) is responsible for the appearance of cap cells in the growing terminal end bud (TEB) that in turn give rise to the myoepithelial lineage of the subtending mammary ductal system. This same clone is shown among the body cells of the marked TEBs as a group of closely associated cells also marked by lacZ. We conclude that this clone found in the body of the TEB gives rise to the specialized cap cells and subsequently to the myoepithelial lineage of the subtending mammary ducts. This is a slightly different interpretation than that presented by Williams and Daniel in 1983 [4], who postulated that the cap cells themselves were multipotent and gave rise to both luminal and myoepithelial progeny in the growing ductal system. It is clear from our work that none of the luminal cells in the subtending ducts bear the unsilenced lacZ-marked X chromosome, therefore we conclude that the luminal cells arise from a different set of progenitors. These conclusions mesh very well with our earlier discovery that lobule-only and duct-only progenitors are present amongst nulliparous mammary epithelium during transplantation of limiting dilutions of epithelial cells from primary cultures of non-pregnant females [14]. In addition this finding was confirmed during serial passages of mammary tissue into subsequently impregnated hosts where ductal and lobular development were shown to be lost independently from one another [2].
